# The first Saudi lung cancer guidelines

**DOI:** 10.4103/1817-1737.43155

**Published:** 2008

**Authors:** John Howington

**Affiliations:** *Department of Surgery, Northwestern University Feinberg School of Medicine, Evanston Northwestern Healthcare, Evanston, IL, USA*

Cancer is a leading cause of death worldwide: it accounted for 7.9 million deaths (around 13% of all deaths) in 2007. Tobacco use is the single most important risk factor for cancer, especially lung cancer. Lung cancer accounts for 1.4 million deaths per year, making it the number one cancer killer in both men and women worldwide.

In 2002 lung and bronchial cancer were the third leading cause of death in men and the tenth leading cause of death in women in Saudi Arabia.[1] Since 1970 the prevalence of smoking has increased in Saudi Arabia, as in the rest of the world, and this will likely lead to a lung cancer epidemic in the coming decades.

Recent European studies have shown that variations in lung cancer care can negatively impact survival in a population.[2] These studies highlight the need for a consistent and less varied approach to lung cancer treatment. While guidelines are not intended to be a recipe for patient care, they do provide a framework for the approach to lung cancer patients with specific types of cancer (small cell or non-small cell) and the evidence behind a recommended workup and/or treatment of lung cancer patients at various clinical stages.

There is an ever-expanding body of knowledge in medicine. It is difficult, if not impossible, for the individual practicing physician to master this body of knowledge by himself or herself. Cancer guidelines assist the physician in assimilating and evaluating the evidence for the current best practice in the care of the lung cancer patient and in implementing it in their clinical practice.

The process involved in guideline development has steadily advanced and now embraces more rigorous evidence standards, multidisciplinary participation, and a patient-centered approach. These advances have been incorporated into these lung cancer management guidelines.

While the American College of Chest Physician, National Comprehensive Cancer Center, and other lung cancer guidelines exist, regional guidelines better take into account the region-specific epidemiology, the available technologies (for imaging, diagnosis, and staging), and the therapeutic options available in the region. Regional guidelines also take into consideration the financial and resource limitations and the social values of the society in which the guidelines are implemented.

The lung cancer guidelines that appear as a supplement to this issue of the journal were developed by internationally trained physicians from various medical and surgical disciplines working in the field of thoracic oncology at leading medical institutions in the Kingdom of Saudi Arabia. The committee members also represent the Saudi Oncology Society and the Saudi Thoracic Society.

The evidence in these guidelines is rated at three levels. EL-1 (highest level) evidence involves data from phase 3 randomized trials or meta-analyses. EL-2 (intermediate-level) evidence is based on good phase 2 trials or phase 3 trials with limitations. EL-3 (low-level) evidence is based on retrospective or observational data and/or expert opinion. This easy-to-follow grading system allows the reader to accurately assess the applicability of the guideline in individual patients.

This evidence-based guideline is a comprehensive overview and covers the epidemiology, radiographic workup, pathology specimen handling and reporting, staging (including discussion of the changes coming with the seventh edition of the staging system), initial evaluation, surgical management, radiation therapy, and systemic therapy for lung cancer patients.

The lung cancer management guidelines are stage specific and easy to follow and incorporate the current state of the art in the treatment of lung cancer patients. The guidelines are supported by well-written and well-researched manuscripts.

The lung cancer management guidelines are also concise and practical and, at two pages, could easily be posted in areas of care to facilitate their implementation and assure best practices. These lung cancer guidelines will result in further improvements in the treatment and outcomes of lung cancer patients in the Kingdom of Saudi Arabia.
